# Impact of employment on the elderly in a super-aging society during the COVID-19 pandemic in Japan

**DOI:** 10.1038/s41598-023-45270-5

**Published:** 2023-10-30

**Authors:** Masakazu Imaoka, Fumie Tazaki, Mitsumasa Hida, Ryota Imai, Hidetoshi Nakao, Takao Inoue, Jyunya Orui, Misa Nakamura

**Affiliations:** 1 Department of Rehabilitation, Osaka Kawasaki Rehabilitation University, 158 Mizuma, Kaizuka, Osaka 597-0104 Japan; 2 Cognitive Reserve Research Center, Kaizuka, Osaka Japan; 3 Department of Comprehensive Rehabilitation, Graduate School, Osaka Prefecture University, Sakai, Osaka Japan; 4 Department of Preventive Gerontology, National Center for Geriatrics and Gerontology, Obu, Aichi Japan; 5 Department of Physical Therapy, Faculty of Welfare, Josai International University, Togane, Chiba Japan

**Keywords:** Psychology, Health care

## Abstract

Employment of the elderly is gaining importance in Japan’s super-aging society. However, investigating the role of employment on the health of the elderly population during the coronavirus disease 2019 (COVID-19) pandemic, wherein they were susceptible, is necessary. We aimed to investigate whether the presence or absence of employment affected motor and cognitive functions in the elderly during the COVID-19 pandemic. The study involved 144 individuals aged ≥ 65 years who participated in the medical examination project from August to September 2021. The participants were divided into employed and non-employed groups. The motor function was evaluated by determining the walking speed, skeletal muscle mass, 2-step test, and bone density. Cognitive function was evaluated using the Mini Mental State Examination and Trail Making Test-A/B (TMT-A/B). For statistical examination, univariate analysis and logistic regression analysis were performed using significantly differential variables. Out of the 144 participants, 33 (22.9%) and 111 (77.1%) were in the employed and non-employed groups, respectively. TMT-A had an odds ratio of 0.96 (95% confidence interval 0.94–0.99) and was an independent factor in the employed group. In conclusion, the attention function was significantly higher in the employed group.

## Introduction

With the advent of a super-aging society in Japan, expectations for the elderly to be employed are increasing yearly^1^. The population of workers aged ≥ 65 years in Japan is increasing, and the proportion of the total labor force aged ≥ 65 years has tripled from 4.9% in 1980 to 12.4% in 2017^2^. In addition, the employment rate of the elderly is 51.0% for those aged 65–69 years, 33.1% for those aged 70–74 years, and 10.5% for those aged ≥ 75 years. It is predicted that the employment rate of the elderly in Japan will increase in the future^3^.

Employment of the elderly is being proposed to supplement the decreasing working-age population and to curb the increasing social security costs^4^. From the perspective of care prevention, employment of the elderly leads to their participation in social activities and contributes to the maintenance of their daily activities^5^. In addition, reduced cognitive decline and risk of death have also been associated with employment of the elderly^6–8^. Moreover, participating in paid employment provides a sense of fulfillment—derived from contributing to the society—for the elderly.

Nonetheless, through an 8-year prospective study on the elderly, Fujiwara et al.^9^ demonstrated that working would be helpful in maintaining basic activities of daily living only in males. A study that analyzed data from the Survey of Health, Aging and Retirement in Europe reported^10^ that retirement improved the health index. However, a Chinese study reported that mental health deteriorates when employment is resumed after retirement, and there is no consensus on the impact of employment on the elderly^11^. There are various reasons for working and different working styles. It is said that the labor force is unlikely to decline in organizations that have flexible working styles and maintain occupational safety and health among the working elderly^12^.

With the rise of coronavirus disease 2019 (COVID-19) caused by severe acute respiratory syndrome coronavirus 2 (SARS-CoV-2) in February 2020, the employment situation of the elderly has changed^13^. The infectious disease has been declared a pandemic. Because the severity of illness and risk of mortality increase with age, the elderly have refrained from going out during the pandemic^14^. According to an investigation^15^ conducted during the COVID-19 emergency, the physical activity of the elderly living in the study area decreased by an average of 26.5%. There is a strong concern that the amount of activity will decrease and the risk of poor health will increase if the elderly refrain from going out^16^. Maintaining high physical and intellectual activity levels reduces the risk of frailty. In other words, being involved in social activities such as employment may have a positive impact on the elderly’s health during pandemic.

However, it remains unclear whether continuing employment during the pandemic has beneficial effects on motor and cognitive functions, as compared with those during normal times. Therefore, the present study aimed to analyze whether the presence or absence of employment affects motor and cognitive functions in individuals aged ≥ 65 years during the pandemic.

## Methods

### Trial design and participants

**Figure 1:**
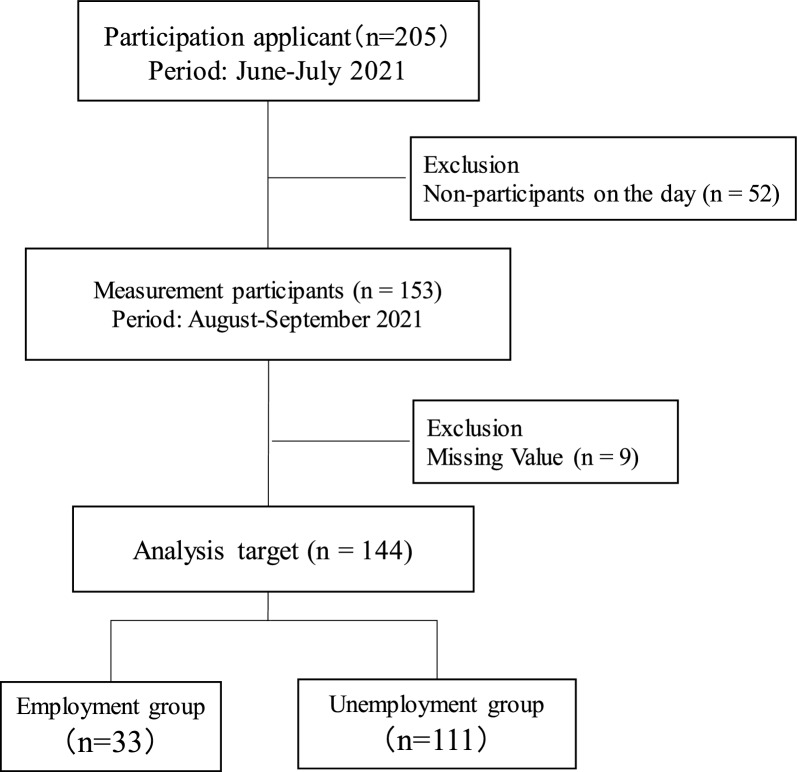
Study flowchart.

Participants aged ≥ 65 years were recruited from among 153 people who underwent health check-ups conducted for a total of six days at three locations in Kaizuka City, Osaka Prefecture (namely, Health and Welfare Center, Yamate District Public Hall, and Hamate District Public Hall) from August to September 2021. A total of 144 participants (mean age: 76.2 ± 5.5 years) were recruited after obtaining informed consent (Fig. 1). Exclusion criteria were as follows: those who were stopped from exercising by a doctor and those with missing measurement data.

### Methodology

The analysis was performed by dividing the participants into two groups. We conducted a survey by questioning whether or not the participants were employed with income. Those who replied in the affirmative were included in the “employed” group, whereas those who replied in the negative were included in the “non-employed” group.

The parameters studied in the survey were grip strength^17^, 2.4-m walking^18^, skeletal muscle mass index (SMI)^18^, bone density^19^, 2-step test^20^, Mini Mental State Examination Japanese Version (MMSE-J)^21,22^ as the general cognitive function test, Trail Making Test-A/B Japanese Version (TMT-A/B) as the attention/execution function test^23,24^, and the revised Japanese version of the Cardiovascular Health Study criteria (revised J-CHS)^25^.

In the 2.4-m walking test, the average walking speed (m/s) was calculated by measuring the time required to walk to and fro on a path with a preliminary track length of 2 m. The participants were instructed to walk at their usual speed. The grip strength of the dominant hand was measured using a grip strength meter (Takei Kikai Kogyo, TKK5401). All measurements were obtained in the standing position. The skeletal muscle mass of the limbs was measured using a body composition meter (InBody-270; InBody, Tokyo, Japan) using the bio-impedance method. The skeletal muscle mass of the limbs was normalized by the square of the height to estimate the SMI. The bone mineral density of the right heel was measured using an ultrasonic bone mineral density measuring device (AOS-100 manufactured by Hitachi, Ltd. Tokyo, Japan) and was compared with the average value of young adults.

For cognitive function, general cognitive function was measured by the MMSE-J and the attention function (selectivity, transsexuality, and distributability) was measured by the TMT-A/B-J. Each survey was conducted individually by volunteer staff and occupational therapists on a one-to-one basis with the participants. Cognitive function tests were conducted in partitioned areas, and occupational therapists, who were in charge of taking breaks and leading booth sections during each test, made rounds to observe participants to minimize the effects of fatigue and inattention on the cognitive function test results.

Components of frailty in the J-CHS were defined as follows: weight loss, weakness, exhaustion, low activity level, and slowness. Weight loss was determined using the question “Have you lost 2 kg or more in the past 6 months?” taken from the Kihon Checklist, which is a self-reporting, comprehensive health checklist. Exhaustion was determined based on a response of “yes” to another question from the Kihon Checklist: “In the last 2 weeks, have you felt tired for no reason?” Weakness was defined as low muscle strength based on the grip strength assessment described earlier. Low activity level was defined as a negative response to the following questions: “Do you engage in moderate levels of physical exercise or sports aimed at health?” and “Do you engage in low levels of physical exercise aimed at health?” Slowness was defined as a walking speed of < 1.0 m/s. Participants were surveyed on the number of the 5 frailty items they possessed.

In the statistical examination, after confirming the normality of the two groups, a univariate analysis was performed using the Mann–Whitney U test. The parameters for which a significant difference was found were the independent variables. “Age” and “sex” were regarded as the adjustment variables, whereas “employment” was considered as the dependent variable. A logistic regression analysis was performed using the forced input method with significantly differential variables. All analyses were performed using SPSS version 28.0 (IBM). For the employment group, a sub-analysis using simple tabulation survey was conducted to determine the reasons for working and the working style. A *p*-value of < 0.05 was considered statistically significant.

### Ethical approval and consent to participate

This study was approved by the Research Ethics Review Committee of Osaka Kawasaki Rehabilitation University (approval ID: OKRU-RA0005). All procedures performed complied with the relevant guidelines and regulations. This study followed the applicable law and was conducted in accordance with Good Clinical Practice guidelines and with the ethical standards laid down in the 1964 Declaration of Helsinki and its later amendments.

## Results

Among the two groups, 33 (22.9%) and 111 (77.1%) participants were employed and unemployed, respectively. Table 1 shows the age, sex, and basic attributes of the participants. The survey parameters showing significant differences in the univariate analysis were grip strength, SMI, TMT-A, and TMT-B. Table 2 shows the results of comparisons between the employed and unemployed groups.

**Table 1:** Characteristics of the study participants.

Parameters	All				Employed group				Unemployed group			*p*-value
	*n* = 144				*n* = 33				*n* = 111			
Age (years)	76.2 ± 5.5				74.5 ± 4.9				77.1 ± 5.8			0.01
Number of female participants (%)	104 (72.2)				16 (48.5)				88 (78.4)			0.03
Height (cm)	154.8 ± 8.1				158.4 ± 8.0				153.8 ± 7.9			0.00
Weight (kg)	53.9 ± 8.9				55.4 ± 8.5				53.4 ± 9.0			0.26

Numerical value: median ± standard deviation or *n* (%).

**Table 2:** Outcome measurements in the two groups.

Parameters	All	Employed group	Unemployed group			*p*-value
	*n* = 144	*n* = 33	*n* = 111			
Gait speed (m/sec)	1.35 ± 0	1.36 ± 0.2	1.35 ± 0.2			0.88
Grip strength (kg)	23.9 ± 7.2	28.3 ± 7.3	22.5 ± 6.7			0.00
SMI (kg/m^2^)	5.97 ± 0.9	6.38 ± 0.92	5.85 ± 0.9			0.00
2-step test (m/m)	1.17 ± 0.19	1.21 ± 0.19	1.15 ± 0.19			0.14
Frail items (score)	0.7 ± 0.9	0.5 ± 0.8	0.7 ± 0.9			0.21
Bone density (%)	86.5 ± 10.9	89.2 ± 12.8	85.6 ± 10.2			0.15
MMSE (score)	28.4 ± 2.3	28.5 ± 2.1	28.4 ± 2.3			0.78
TMT-A (sec)	62.8 ± 26.5	50.4 ± 15.7	66.4 ± 27.9			0.00
TMT-B (sec)	110.5 ± 67.5	88.5 ± 45.7	117.0 ± 71.6			0.01

Numerical value: median ± standard deviation or *n* (%).

*SMI* skeletal muscle mass index, *MMSE* Mini Mental State Examination, *TMT* trail making test.

Table 3 presents results of the logistic regression analysis using the forced input method, with employment as the dependent variable, Grip strength SMI and TMT-A/B as the independent variables, and age and sex as the adjustment variables. The odds ratio (OR) for TMT-A was 0.96 (95% CI 0.94–0.99). The employment group spent significantly less time on the TMT-A, suggesting higher attentional function.

**Table 3:** Odds ratio of factors related to employment (adjusted model).

	OR	95% CI			*p*-value
Grip strength	1.08	0.99 –1.19			0.11
SMI	1.15	0.61–2.18			0.67
TMT-A	0.97	0.94–0.99			0.01
TMT-B	0.99	0.98–1.00			0.11

*OR* odds ratio, *CI* confidence interval (adjusted for age and sex), *SMI* skeletal muscle mass index, *TMT* trail making test.

Regarding the reason for working, 10 participants (30.3%) claimed that they worked for health, 5 (15.2%) for social connection, 5 (15.2%) for income, 4 (12.1%) for survival, 3 (9.1%) for additional income, 2 (6.1%) for having plenty of time to spare, and 4 (12.1%) for other reasons. Table 4 shows the reasons for working by the 33 people in the employed group. Regarding the working style, 1 (3.0%) was a full-time employee, 18 (54.5%) were part-time employees, 8 (24.2%) were self-employed, and 6 (18.2%) were categorized as other working styles (Online Resource 1).

**Table 4:** Classification of reasons for working in the employed group.

Items	*n* = 33	
For health	10	(30.3)
For social connection	5	(15.2)
For living income	5	(15.2)
For survival	4	(12.1)
For additional income	3	(9.1)
I have plenty of time	2	(6.1)
Others	4	(12.1)

Numerical value: *n* (%).

## Discussion

Out of the 144 participants analyzed in this study, 33 (22.9%) were in the employed group, whereas 111 (77.1%) were in the non-employed group. Even after adjustment for age and sex, the attention function was significantly higher in the employed group than in the non-employed group (OR: 0.96, 95% CI 0.94–0.99).

The attention function includes four elements: persistence, selection, transfer, and distribution. However, each element is interrelated^26,27^. In addition, the classification of the attention function is not standardized^28^. Therefore, it is challenging to examine which element of the attention function has an effect. However, the TMT-A used in this study includes visual reading ability, graphomotor speed (correct writing speed), and visual motion processing speed in addition to the four elements of the attention function^29^. It is proposed that since attentional functions are required for the routine execution of tasks while performing jobs, the employed group showed better attention function than the non-employed group. Similarly, there are many opportunities to process visual information and for writing while carrying out jobs. This may be attributed to improved visual reading ability and graphomotor speed depending on the type of job.

In the univariate analysis, significantly higher grip strength and SMI values in the working group could be due to the skewed male–female ratio between the two groups. In this study, 16 out of 33 participants in the employed group were females (48.5%), whereas 87 out of 111 participants in the unemployed group (78.4%) were females. In 2018, the employment rate of the elderly by sex had been reported to be 33.2% for males and 17.4% for females, which is almost a 2:1 ratio^30^. According to a Cabinet Office survey, the employment rate for men over the age of 15 years in Japan is 69.4% for men and 53.0% for women; this rate is slightly higher for men, but it is not as large as the difference in employment rates between men and women among the elderly^31^. Hence it is clear that the working scenario of males and females in Japan differs greatly between the younger and elderly generations.

In the case of other motor functions, there were no differences in walking speed, locomotive 2-step test, and frailty between the two groups. Because the type and content of employment varied, it could be speculated that the motor function was affected by the work content. In particular, of the 33 employees in the working group, 1 (3.0%) continued to work as a full-time employee, suggesting that the daily employment characteristics had little effect on motor function. In addition, a previous study reported that working individuals were associated with pre-frailty^32^. However, this study found no difference in the number of frail participants between the two groups. The reason for this could be the average age of the participants, which is about 3 years younger than in previous studies.

The most common reason for working was found to be health (30.3%). In contrast, other surveys^33^ showed that the majority were working for financial reasons. The difference could be due to the participants of this study, who were interested in getting examined for motor and cognitive functions, and the recruitment was limited to those who could engage for about 1 h and 30 min during the daytime on weekdays. Therefore, it is difficult to generalize and examine the reasons for employment.

There are some limitations to this study. First, it was not possible to establish a causal relationship between doing paid work and the decline in attention function for a cross-sectional survey. Hence, we are conducting a follow-up survey to verify whether the attention function could be kept high by working. Second, the investigation was carried out in an emergency scenario due to the pandemic. Third, the sample size was small, and additional research is needed to generalize the results. Fourth, the lack of information on underlying diseases related to motor and cognitive function does not rule out the effects identified herein. Furthermore, although age was adjusted for in the final multivariate analysis, the effect of aging must be considered as a limitation of this study, as there is an age difference of approximately 2.6 years between the two groups.

However, the impact on employment due to the emergency scenario is still unclear. Therefore, we plan to re-examine the participants and analyze the details starting from 2022.

## Conclusion

The study showed that the elderly in a super-aging society who were employed during the COVID-19 pandemic had high attention function. No effect of employment on motor function and other general cognitive functions was confirmed. This study will help in the better management of the pandemic situation and lead to the improvement in the quality of life among the elderly. Furthermore, the findings herein may provide basic data for intervention studies and other research to maintain and improve attention function in the elderly.

### Supplementary Information

Supplementary Information.

## Data Availability

The datasets used and/or analyzed in the current study are available from the corresponding author on reasonable request.
