# Supplementary material for: Impact of employment on the elderly in a super-aging society during the COVID-19 pandemic in Japan
Source: Sci Rep. 2023 Oct 30;13:18564. doi: 10.1038/s41598-023-45270-5 (PMC10616061; doi:10.1038/s41598-023-45270-5)
Supplement: Supplementary file 1 — Supplementary Information. [file 41598_2023_45270_MOESM1_ESM.docx]

| **Online Resource 1** Working style | | |
| --- | --- | --- |
| Classification | *n*=33 | |
| Full-time employee | 1 | (3.0) |
| Part-time job | 18 | (54.5) |
| Self-employment | 8 | (24.2) |
| Others | 6 | (18.2) |
| Numerical value: *n* (%) | | |
